# Mice Cohabiting With Familiar Conspecific in Chronic Stress Condition Exhibit Methamphetamine-Induced Locomotor Sensitization and Augmented Consolation Behavior

**DOI:** 10.3389/fnbeh.2022.835717

**Published:** 2022-04-18

**Authors:** Paulo Eduardo Carneiro de Oliveira, Isabela Miranda Carmona, Mariana Casarotto, Lara Maria Silveira, Anna Cecília Bezerra Oliveira, Azair Canto-de-Souza

**Affiliations:** ^1^Psychobiology Group/Department of Psychology/CECH – Federal University of São Carlos, São Carlos, Brazil; ^2^Joint Graduate Program in Physiological Sciences UFSCar/UNESP, Federal University of São Carlos, São Carlos, Brazil; ^3^Graduate Program in Psychology, Federal University of São Carlos, São Carlos, Brazil; ^4^Neuroscience and Behavioral Institute, Ribeirão Preto, Brazil

**Keywords:** cross-sensitization, anxiety, consolation, methamphetamine, emotional contagion, mice, restraint stress, familiarity

## Abstract

Recognizing and sharing emotions are essential for species survival, but in some cases, living with a conspecific in distress condition may induce negative emotional states through empathy-like processes. Studies have reported that stressors promote psychiatric disorders in both, those who suffer directly and who witness these aversive episodes, principally whether social proximity is involved. However, the mechanisms underlying the harmful outcomes of emotional contagion need more studies, mainly in the drug addiction-related behaviors. Here, we investigated the relevance of familiarity and the effects of cohabitation with a partner submitted to chronic stress in the anxiety-like, locomotor sensitization, and consolation behaviors. Male Swiss mice were housed in pairs during different periods to test the establishment of familiarity and the stress-induced anxiety behavior in the elevated plus maze. Another cohort was housed with a conspecific subjected to repeated restraint stress (1 h/day) for 14 days. During chronic restraint the allogrooming was measured and after the stress period mice were tested in the open field for evaluation of anxiety and locomotor cross-sensitization induced by methamphetamine. We found that familiarity was established after 14 days of cohabitation and the anxiogenic behavior appeared after 14 days of stress. Repeated restraint stress also increased anxiety in the open field test and induced locomotor cross-sensitization in the stressed mice and their cagemates. Cagemates also exhibited an increase in the consolation behavior after stress sessions when compared to control mice. These results indicate that changes in drug abuse-related, consolation, and affective behaviors may be precipitated through emotional contagion in familiar conspecifics.

## Introduction

Empathy is the ability to share emotions where the subject takes the perspective of the object, and this phenomenon generates the same affective states (Preston and de Waal, 2002; de Waal, 2008). Although this is evolutionarily essential for species survival, this process may be as deleterious as a direct experience to aversive stimuli since it induces similar autonomic and behavioral responses (de Waal, 2008; Lamm et al., 2011; Panksepp and Lahvis, 2011; Benuzzi et al., 2018). Thus, witnessing a conspecific in suffering conditions could drive negative emotional states (Pfefferbaum et al., 2000; Blanchard et al., 2004; Langeland et al., 2004; Feinstein et al., 2013).

Due to its importance, animal models have been developed to understand the neurobiological basis of empathy-like behaviors, such as emotional contagion, consolation, and helping activity (Knapska et al., 2006; Langford et al., 2006; Bartal et al., 2011; Mogil, 2015; Burkett et al., 2016; Karakilic et al., 2018). Studies from our laboratory, for example, observed anxiogenic-like behaviors and anhedonia in mice living with a conspecific subjected to chronic restraint stress (Carneiro de Oliveira et al., 2017) and neuropathic conditions (Baptista-de-Souza et al., 2015, 2021; Carmona et al., 2016; Benassi-Cezar et al., 2020). In this sense, through emotional contagion, researchers have emphasized that distress-induced psychiatric disorders, such as anxiety and depression, can occur vicariously.

Besides anxiety- and depression-like disorders, drug abuse is a relapsing psychiatric illness frequently associated with stress conditions (Piazza et al., 1990; Koob et al., 2014; Moal, 2016; Camarini et al., 2018), in which an important process involved is the cross-sensitization. Therefore, drug effects become successively greater after chronic exposure to stressors. A clinical study conducted by Booij et al. (2016) reported cross-sensitization between amphetamine and stress through the observation of enhanced physiological parameters such as anxiety, cortisol, and heart rate. Moreover, chronic exposure to several types of stress, such as restraint (Deroche et al., 1992; Kabbaj et al., 2002; Lepsch et al., 2005; Doremus-Fitzwater and Spear, 2010; Cruz et al., 2012; Carneiro de Oliveira et al., 2016), footshock (Cheng et al., 2020), and social defeat (Nikulina et al., 2004, 2012; Yap et al., 2005; Johnston et al., 2015; Rudolph et al., 2020) induces behavioral cross-sensitization in rodents. Thus, despite not being deeply explored, psychological stress may cause drug abuse-like behaviors similar to those who experienced stress directly. For instance, studies demonstrated increased rewarding effects of cocaine and alcohol in animals that witnessed chronic defeat stress applied to their cagemates (Garcia-Carachure et al., 2020; Barchiesi et al., 2021). However, no studies have verified the consequences of vicarious stress in drug-induced locomotor sensitization.

Additionally, it is relevant to highlight the role of familiarity in empathy-related behaviors when the subjects face the object in suffering. Preclinical studies found increased emotional responses in siblings, sexual mates, and cagemates, but not strangers, witnessing the other in a distressful situation (Langford et al., 2006; Jeon et al., 2010; Gonzalez-Liencres et al., 2014; Martin et al., 2015; Lidhar et al., 2017; Pisansky et al., 2017; Lu et al., 2018). For example, in humans, there is a higher prevalence of alcohol/drug abuse in family members and caregivers of patients with long-lasting disturbances compared to age-matched individuals coexisting with healthy individuals (Gallant and Connell, 1997; De Bellis et al., 2001; Bayen et al., 2014; Rumpold et al., 2016; Webber et al., 2020). This interpersonal proximity is also required for another empathy-related behavior, the consolation. Consolation is a prosocial behavior in which an uninvolved spectator expends affiliative contact toward a distressed conspecific aiming for a calming effect (Preston and de Waal, 2002; de Waal, 2008). Research has shown increased allogrooming, an analogous behavior to consolation in rodents, toward a familiar experience of pain (Li et al., 2018; Lu et al., 2018; Du et al., 2020), social defeat stress (Li et al., 2019), and footshock (Knapska et al., 2010; Burkett et al., 2016; Kiyokawa et al., 2019). However, these studies evaluated allogrooming in cases of acute stress, but not during chronic stress.

For this purpose, in the present study, we investigated the time-period necessary for the formation of pair bound through the exhibition of anxiety-like behavior in the elevated plus maze. We also assessed whether cohabitation with a conspecific subjected to repeated restraint stress may provoke anxiety and locomotor cross-sensitization induced by methamphetamine in the open field test. Finally, we quantified the allogrooming behavior after the stress sessions on three different days to evaluate the influence of chronic stress on consolation-like behaviors.

## Materials and Methods

### Subjects and Ethics

In this study, 455 male, 21-day-old, Swiss mice (18–20 g) obtained from the animal breeding facility of the Federal University of São Carlos, São Paulo, Brazil, were moved to the animal facility of the Psychobiology Group laboratory. After 1 week of habituation, mice were housed in two per cage [19 cm (width) × 30 cm (length) × 14 cm (height), cage floor covered with sawdust]. Mice were maintained under a regular light–dark cycle (12 h/12 h, lights on at 07:00) and controlled temperature (24°C ± 1°C) with unrestricted access to food and water, except during the brief test periods. The experiments were conducted during the light phase between 09:00 and 17:30. All procedures were performed in accordance with the recommended protocol approved by the Brazilian Guidelines for Care and Use of Animals for Scientific and Educational Purposes, elaborated by the National Council of Control of Animal Testing (CONCEA). This study was approved by the Ethics Committee on Animal Experiments (CEUA 4996150816).

### Drugs

Methamphetamine was dissolved in saline (0.9% NaCl) and injected intraperitoneally at a dose of 1.5 mg/kg for the cross-sensitization experiment. The doses were based on a pilot study.

### Restraint Stress

Chronic restraint stress was induced using a PVC tube [14 cm (length) × 3 cm (diameter)]. One of the animals (stress) was placed inside the tube 1 h a day in its housing box in the presence of its conspecific (cagemate) in an adjacent room. Animals from the control group were transferred to another adjacent room during the stress period (Carneiro de Oliveira et al., 2017).

### Body Weight Gain

To assess whether restraint was effective, all subjects were weighted after the first and last stress sessions (15th and 28th day; see Experimental Procedures for details). Weight gain was calculated based on the equation [(weight on 28th day) − (weight on 15th day)] (Carneiro de Oliveira et al., 2017).

### Elevated Plus-Maze

The Elevated Plus-Maze (EPM) test assessed anxiety-like behaviors. The EPM used was similar to that described by Lister (1987) and consisted of a wooden maze coated by plastic laminate, raised 38.5 cm from the floor, with four arms arranged in a plus format with two opposite arms closed by transparent glass walls (30 × 5 × 15 cm), connected by a common central platform (5 × 5 cm) with two opposite open arms (30 × 5 × 0.25 cm). All the tests were conducted during the light phase of the light–dark cycle under the illumination of 77 lux on the floor of the apparatus (Carneiro de Oliveira et al., 2017). The animals were placed in the center of the maze facing an open arm. The number of entries and the time spent in each arm were recorded for 5 min. An entry was considered when the animal placed all four paws into an arm. Conventional measures were the percentage of open arm entries (%OE) [(open/total entries) × 100] and the percentage of time spent in open arms [(open/total time) × 100]. These activities have been used as an index of anxiety behavior (Lister, 1987; Rodgers and Johnson, 1995). The number of closed-arm entries (CEs) was used to measure locomotor activity in mice. Complementary behaviors measured were the percentage of the time spent in the central platform [(central/total time) × 100], the number of head-dippings (exploratory movement of head/shoulders over sides of the maze), percentage of protected head-dipping [(protected/total) × 100], the number of stretch-attend postures (SAP; an exploratory posture in which the mouse stretches forward and retracts to the original position without locomotion), and the percentage of protected SAP [(protected/total) × 100]. Behaviors such as head-dipping and SAP were used to measure risk assessment (Rodgers and Johnson, 1995). Depending on where these behaviors were exhibited, they were counted as protected or unprotected. In line with previous studies, the closed arms and central platform were together designated as protected areas of the maze, while the open arms were designated as unprotected areas (Rodgers and Johnson, 1995). Between tests, the apparatus was cleaned with ethanol 20% and dried with a cloth. All sessions were recorded using a vertically mounted camera linked to a computer for the posterior analysis. Test videos were scored by a highly trained observer using the free software package X-PloRat (Tejada et al., 2017).

### Open Field Test

All mice were tested in an open field for 5 min to assess anxiety. The first contact of a rodent with an open arena was used to evaluate the emotional variations induced by a novel environment. In this sense, animals exhibit a behavior called thigmotaxis, or a tendency to stay close to the walls avoiding unknown open areas (Treit and Fundytus, 1988; Gould et al., 2009). On the test day, all groups were exposed to an opaque plastic arena with a dark floor [41 cm (length) × 34 cm (width) × 16 cm (height); center zone: 24.6 cm (length) × 20.4 cm (width)]. The apparatus was cleaned with 20% ethanol for each test. The behaviors were recorded using video equipment, and the following parameters were analyzed: number of entries in the center zone (EC), total time spent in the center zone in seconds (TC), percentage of time spent in the center zone (%TC), distance traveled in the center zone in meters (DC), percentage of distance traveled in the center zone (%DC), and total locomotor activity. The exploration of the center zone was used as an index of anxiety-like behavior (Archer, 1973; Gould et al., 2009) and total locomotor activity was used to verify some motor impairment. The behaviors were analyzed using ANY maze software (Stoelting Co., Wood Dale, IL, United States) (Morais-Silva et al., 2016a,b).

### Locomotor Sensitization

On the test day, all groups were exposed to an opaque plastic arena with a dark floor [41 cm (length) × 34 cm (width) × 16 cm (height)], which was the same as that used for OFt. The animals were placed in the center of the arena and allowed to move freely for 15 min (900 s) for habituation to the open field. The first 5 min of habituation was used to evaluate anxiety-like behaviors, as described above (item 2.7). After OFt, the animals remained in the arena, and the locomotor activity was measured as the habituation period. At the end of the habituation, the mice were removed from the arena, received an intraperitoneal saline injection (100 μL/10 g body weight), and returned to the open field for more than 30 min. After saline, mice were injected with methamphetamine (1.5 mg/kg, i.p.) and the locomotor activity was measured for 60 min (Leão et al., 2013; Whitaker et al., 2016). The habituation, saline, and methamphetamine periods were recorded using video equipment for post-analysis. The open field was cleaned with 20% ethanol between each animal. Locomotor activity was measured using the ANY-maze software (Stoelting Co., Wood Dale, IL, United States) (Morais-Silva et al., 2016a,b).

### Consolation-Like Behavior

Consolation behavior parameters were latency of allogrooming onset, time spent performing allogrooming, time spent doing self-grooming, and percentage of animals that exhibited allogrooming behavior on each assessed day. Allogrooming consists of rhythmic licks or rubs with the paws of another animal body or head. Grooming directed toward the rear (anogenital, genital, or tail) was excluded (Burkett et al., 2016; Li et al., 2019; Du et al., 2020).

### Experimental Design

#### Influence of Familiarity in Anxiety-Like Behaviors of Mice Tested in the Elevated Plus-Maze

**FIGURE 1:**
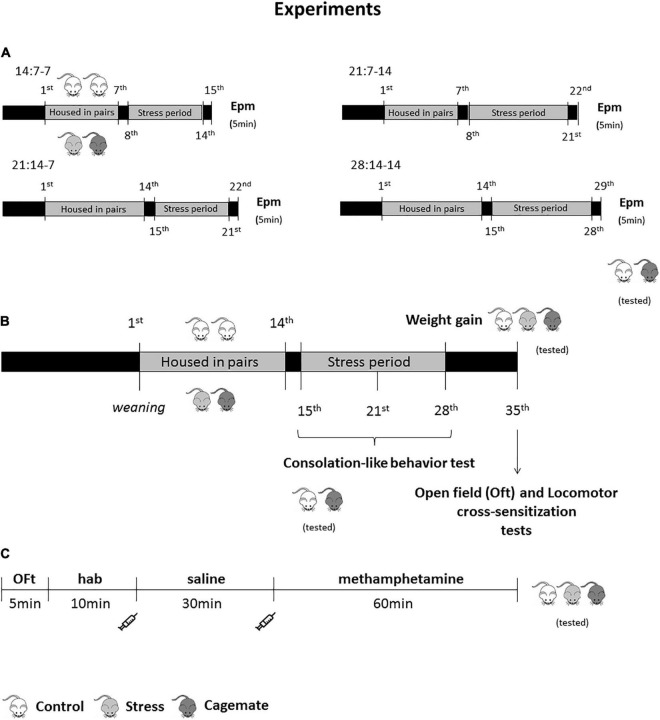
Schematic representation of the experimental protocol. **(A)** Procedure performed for the test of the time-period required to establish the familiarity and to induce anxiety through chronic stress (tested groups: control and cagemate); **(B)** timeline regarding the procedure used to evaluate consolation-like behavior (tested groups: control and cagemate), weight gain (tested groups: control, stress, and cagemate), anxiety-like behavior in the OFt and locomotor cross-sensitization induced by methamphetamine; **(C)** timeline referring to open field and cross-sensitization in the test-day (tested groups: control, stress, and cagemate).

In previous studies from our laboratory, we investigated the consequences of living with a conspecific subjected to chronic restraint stress (Carneiro de Oliveira et al., 2017) or chronic neuropathic pain (Baptista-de-Souza et al., 2015, 2021; Carmona et al., 2016; Zaniboni et al., 2018; Benassi-Cezar et al., 2020; Tavares et al., 2021) for 14 days. In these studies, mice were kept in dyads for 14 days before the beginning of stress sessions or sciatic nerve constriction to establish familiarity, as proposed by Langford et al. (2006). In this context, the protocols lasted 28 days in which in the first 14 days, mice developed familiarity, and in the next 14 days, mice were chronically exposed to conspecific distress. Here, we assessed temporal differences in familiarity establishment and stress-induced emotional contagion. As described above (see Section “Restraint Stress”), male mice were subjected to repeated restraint stress in the presence of their cages (*n* = 40), and the control group (*n* = 61) was left undisturbed during the stress session. Thus, we had four study periods: 14 total days, 7 days to establish familiarity and exposure to conspecific stress for 7 days [(14:7-7), control (*n* = 17) and cagemate (*n* = 12)]; 21 total days, 7 days to establish familiarity and exposure to conspecific stress for 14 days [(21:7-14), control (*n* = 17), and cagemate (*n* = 9)]; 21 total days, 14 days to establish familiarity and exposure to conspecific stress for 7 days [(21:14-7), control (*n* = 18) and cagemate (*n* = 9)]; 28 total days, 14 days to establish familiarity and exposure to conspecific stress for 14 days [(28:14-14) control (*n* = 9) and cagemate (*n* = 10)]. Twenty-four hours after the last stress session, control, and cagemate groups were tested in the EPM for analysis of anxiety-like behaviors (Figure 1A). The animals were transferred to the experimental room 30 min before the test for acclimatization and then placed in the EPM and allowed to move freely for 5 min (300 s).

#### Assessment of Anxiety-Like Behaviors in the Open Field Test and Evaluation of Locomotor Cross-Sensitization Induced by Methamphetamine Challenge

On the first experiment day (weaning), 128 male mice were housed in pairs for 14 days and left undisturbed until the 14th day, except for cage cleaning. On the 15th day, the animals were divided into two groups: stress, in which one animal of each pair was subjected to restraint stress for 1 h for 14 days; cagemate, an observer that witnessed conspecific exposure to restraint stress and control, in which no animal of the dyad was exposed to restraint stress. Seven days after the last stress session, the mice were submitted to OFt (Figure 1B). The animals were transferred to the experimental room 30 min before the test for acclimatization and then placed in the center of the arena and allowed to move freely for 5 min (300 s), as described in Section “Locomotor Sensitization.” All subjects were weighed on the 15th and 28th days for evaluation of body weight gain. Seven days after the last stress session, as described above (item 2.10.3), 128 male mice from all groups [control (*n* = 44); stress (*n* = 42) and cagemate (*n* = 42)] were submitted to locomotor sensitization test (Figure 1C). The habituation to the apparatus started immediately after the OFt and lasted 10 min (600 s). After the habituation period, mice were injected with saline (100 μL/10 g body weight), and their locomotor activity was measured for more than 30 min. Lastly, the animals were administered methamphetamine (1.5 mg/kg, i.p.) and the distance traveled was estimated for 60 min (3,600 s). After data analysis, the groups were divided (n_*total*_/3 for each condition group) in high-, mid-, and low-response groups considering the distance traveled during the methamphetamine period, generating nine different groups [high response: control (*n* = 15), stress (*n* = 14), and cagemate (*n* = 14); mid-response: control (*n* = 14), stress (*n* = 14), cagemate (*n* = 14); low-response: control (*n* = 15), stress (*n* = 14), cagemate (*n* = 14)].

#### Assessment of Consolation-Like Behaviors

Consolation-like behavior was assessed on the 15th, 21st, and 28th days after the stress sessions. At the end of the stress session, mice were returned to the home cage, and consolation behaviors from the control (*n* = 20) and cagemate (*n* = 21) groups were recorded for 15 min for posterior analysis (Figure 1B). The animals for evaluation of the interaction after the stress sessions were randomly chosen and remained the same on the three days of allogrooming behavior scoring. In the control group, the animal analyzed was the one that exhibited allogrooming.

### Statistical Analysis

All data were initially evaluated for homogeneity of variance (Levene’s test). To determine the influence of familiarity and the number of stress sessions, data were analyzed using two-way ANOVA considering days of cohabitation and days of stress sessions. Although the two-way ANOVA indicated no difference between the groups in almost all evaluated behaviors, we noted that in several cases we could see a visual difference among the groups. Furthermore, statistical analyses revealed the influence of familiarity factor over several behaviors (Table 1). Thus, we decided to analyze the groups separately using Student’s *t*-test for independent samples. Data from body weight gain, OFt, and locomotor cross-sensitization were analyzed using one-way analysis of variance (ANOVA) considering stress factors (with or without stress). For the difference of prevalence of subjects that displayed allogrooming behavior on each day of measurement, considering the control and cagemate groups, we used the Fisher’s exact test. Consolation-like behavior data were subjected to a two-way ANOVA considering stress and day factors. When ANOVA analyses were statistically significant, Newman–Keuls *post hoc* test was applied for comparisons among the groups. Results of statistical tests with *p*-values less than 0.05 were considered significant.

**TABLE 1:** Two-way ANOVA for anxiety-like behavior evaluated in the elevated plus maze.

	Groups			
	14:7-7		21-14-7	
	Control	Cagemate	Control	Cagemate
	
Behavior	Familiarity	Stress		Interaction
Open arm entries (%)	*F*_(1_,_52)_ = 0.47; *p* = 0.50	*F*_(1_,_52)_ = 0.18; *p* = 0.67		*F*_(1_,_52)_ = 0.30; *p* = 0.59
Open arm time (%)	*F*_(1_,_52)_ = 1.84; *p* = 0.18	*F*_(1_,_52)_ = 0.10; *p* = 0.76		*F*_(1_,_52)_ = 0.54; *p* = 0.47
Closed arm entries (frequency)	*F*_(1_,_52)_ = 9.59; *p* < 0.05	*F*_(1_,_52)_ = 6.36; *p* < 0.05		*F*_(1_,_52)_ = 0.21; *p* = 0.65
Center time (%)	*F*_(1_,_52)_ = 0.13; *p* = 0.72	*F*_(1_,_52)_ = 1.90; *p* = 0.17		*F*_(1_,_52)_ = 0.00; *p* > 0.99
SAP (frequency)	*F*_(1_,_52)_ = 14.11; *p* < 0.05	*F*_(1_,_52)_ = 29.64; *p* < 0.05		*F*_(1_,_52)_ = 30.00; *p* < 0.05
Protected SAP (%)	*F*_(1_,_52)_ = 0.03; *p* = 0.88	*F*_(1_,_52)_ = 2.62; *p* = 0.11		*F*_(1_,_52)_ = 0.69; *p* = 0.41
Head-dipping (frequency)	*F*_(1_,_52)_ = 12.37; *p* < 0.05	*F*_(1_,_52)_ = 3.27; *p* = 0.08		*F*_(1_,_52)_ = 4.56; *p* < 0.05
Protected head-dipping (%)	*F*_(1_,_52)_ = 1.67; *p* = 0.20	*F*_(1_,_52)_ = 3.56; *p* = 0.07		*F*_(1_,_52)_ = 7.39; *p* < 0.05

	**21:7-14**		**28:14-14**	

Open arm entries (%)	*F*_(1_,_41)_ = 2.28; *p* = 0.14	*F*_(1_,_41)_ = 2.45; *p* = 0.13		*F*_(1_,_41)_ = 2.15; *p* = 0.15
Open arm time (%)	*F*_(1_,_41)_ = 3.65; *p* = 0.06	*F*_(1_,_41)_ = 0.85; *p* = 0.36		*F*_(1_,_41)_ = 0.85; *p* = 0.36
Closed arm entries (frequency)	*F*_(1_,_41)_ = 5.64; *p* < 0.05	*F*_(1_,_41)_ = 0.58; *p* = 0.45		*F*_(1_,_41)_ = 0.01; *p* = 0.93
Center time (%)	*F*_(1_,_41)_ = 9.30; *p* < 0.05	*F*_(1_,_41)_ = 1.96; *p* = 0.17		*F*_(1_,_41)_ = 0.91; *p* = 0.35
SAP (frequency)	*F*_(1_,_41)_ = 56.55; *p* < 0.05	*F*_(1_,_41)_ = 0.00; *p* = 0.99		*F*_(1_,_41)_ = 0.08; *p* = 0.79
Protected SAP (%)	*F*_(1_,_41)_ = 9.02; *p* < 0.05	*F*_(1_,_41)_ = 1.67; *p* = 0.20		*F*_(1_,_41)_ = 3.07; *p* = 0.09
Head-dipping (frequency)	*F*_(1_,_41)_ = 0.49; *p* = 0.49	*F*_(1_,_41)_ = 5.94; *p* < 0.05		*F*_(1_,_41)_ = 0.09; *p* = 0.76
Protected head-dipping (%)	*F*_(1_,_41)_ = 9.21; *p* < 0.05	*F*_(1_,_41)_ = 9.45; *p* < 0.05		*F*_(1_,_41)_ = 1.85; *p* = 0.18

*The difference was significant for p-value less than 0.05 (p < 0.05).*

## Results

### Emotional Contagion-Induced Anxiety Behavior Is Seen Only After 14 Days of Familiarity and 14 Days of Repeated Stress

Two-way ANOVA indicated no significant effect of familiarity and stress factors as well as interaction (familiarity × stress) in the percentage of entries and the percentage of time spent in the open arms between the groups 14:7-7 (control and cagemate) and 21:7-14 (control and cagemates) (Table 1). Two-way ANOVA also showed no significant effect of familiarity and stress factors as well as interaction (familiarity × stress) in the percentage of entries and the percentage of time spent in the open arms between the groups 21:14-7 (control and cagemate) and 28:14-14 (control and cagemates) (Table 1).

Student’s *t*-test revealed differences in the percentage of open arm entries [*t*_(17)_ = 3.51; *p* < 0.05] and time spent in open arms [*t*_(17)_ = 2.09; *p* = 0.052] only in the 28:14-14 protocol period (Table 2), demonstrating an anxiogenic-like effect induced by 14 days of familiarity, followed by 14 days of witnessing the restraint stress. Regarding the complementary behaviors, 14:7-7 diminished the frequency of total SAP [*t*_(27)_ = 9.19; *p* < 0.05] and augmented the percentage of protected SAP [*t*_(27)_ = −2.75; *p* < 0.05] and total head-dipping [*t*_(27)_ = −2.94; *p* < 0.05] in the cagemate group (Table 2). Protocol periods of 21 days, 21:7-14 [*t*_(24)_ = −2.39; *p* < 0.05] and 21:14-7 [*t*_(25)_ = −2.45; *p* < 0.05], only increased the percentage of protected head-dipping in the cagemate group (Table 2). The protocol period of 28 days (28:14-14) induced an increase in the percentage of time in the center of the EPM [*t*_(17)_ = −2.45; *p* < 0.05], percentage of protected SAP [*t*_(17)_ = −3.68; *p* < 0.05], and percentage of protected head-dipping [*t*_(17)_ = −4.14; *p* < 0.05] of cagemate compared to the respective control group (Table 2). Although the two-way ANOVA had demonstrated no differences among the groups in several behaviors, Student’s *t*-test indicated that anxiogenic-like behaviors were observed only after 14 days to establish pair-bound, followed by 14 days of exposure to vicariously restrained stress sessions.

**TABLE 2:** Student’s *t*-test for anxiety-like behavior evaluated in the elevated plus maze.

	Protocol period											
	14:7-7			21:7-14			21:14-7			28:14-14		
Behavior	Control	Cagemate		Control	Cagemate		Control	Cagemate		Control	Cagemate	
Open arm entries (%)	29.7 ± 3.0	30.4 ± 5.1	*t*_(27)_ = −0.14; *p* = 0.89	40.0 ± 6.6	39.3 ± 9.2	*t*_(24)_ = 0.07; *p* = 0.95	28.9 ± 6.4	23.2 ± 8.0	*t*_(25)_ = 0.54; *p* = 0.60	39.7 ± 4.0	18.6 ± 4.4#	*t*_(17)_ = 3.51; *p* < 0.05
Open arm time (%)	18.7 ± 2.3	23.6 ± 4.7	*t*_(27)_ = −1.01; *p* = 0.32	28.5 ± 6.2	28.5 ± 8.3	*t*_(24)_ = −0.01; *p* = 0.99	15.8 ± 5.3	13.8 ± 5.3	*t*_(25)_ = 0.24; *p* = 0.82	22.1 ± 4.9	10.2 ± 3.1§	*t*_(17)_ = 2.09; *p* = 0.052
Closed arm entries (frequency)	9.1 ± 0.7	10.9 ± 0.8	*t*_(27)_ = −1.82; *p* = 0.08	7.0 ± 0.9	7.7 ± 1.4	*t*_(24)_ = −0.42; *p* = 0.68	6.0 ± 0.6	8.6 ± 1.6	*t*_(25)_ = −1.83; *p* = 0.08	9.4 ± 0.9	10.3 ± 0.8	*t*_(17)_ = −0.68; *p* = 0.51
Center time (%)	37.3 ± 2.2	31.9 ± 2.2	*t*_(27)_ = 1.56; *p* = 0.13	42.6 ± 4.8	44.7 ± 4.7	*t*_(24)_ = −0.28; *p* = 0.78	38.7 ± 4.8	33.3 ± 4.3	*t*_(25)_ = 0.71; *p* = 0.49	23.9 ± 2.8	34.9 ± 3.3#	*t*_(17)_ = −2.45; *p* < 0.05
SAP (frequency)	50.2 ± 2.6	17.2 ± 2.0#	*t*_(27)_ = 9.19; *p* < 0.05	45.4 ± 3.1	46.3 ± 2.8	*t*_(24)_ = −0.17; *p* = 0.87	45.0 ± 3.3	45.1 ± 2.5	*t*_(25)_ = −0.01; *p* = 0.99	20.2 ± 3.7	19.2 ± 3.4	*t*_(17)_ = 0.20; *p* = 0.84
Protected SAP (%)	78.3 ± 2.6	89.2 ± 2.8#	*t*_(27)_ = −2.75; *p* < 0.05	70.9 ± 6.5	68.0 ± 7.0	*t*_(24)_ = 0.26; *p* = 0.80	81.3 ± 5.3	84.8 ± 5.1	*t*_(25)_ = −0.41; *p* = 0.68	78.8 ± 5.3	98.0 ± 1.0#	*t*_(17)_ = −3.68; *p* < 0.05
Head-dipping (frequency)	24.7 ± 2.4	34.4 ± 1.9#	*t*_(27)_ = −2.94; *p* < 0.05	28.7 ± 2.4	37.7 ± 3.9	*t*_(24)_ = −1.77; *p* = 0.09	21.3 ± 1.2	20.5 ± 4.7	*t*_(25)_ = 0.22; *p* = 0.83	27.4 ± 3.6	34.4 ± 3.4	*t*_(17)_ = −1.38; *p* = 0.19
Protected head-dipping (%)	69.9 ± 4.6	63.1 ± 6.1	*t*_(27)_ = 0.90; *p* = 0.38	29.3 ± 8.3	62.9 ± 8.3 #	*t*_(24)_ = −2.39; *p* < 0.05	37.1 ± 10.1	74.8 ± 6.7#	*t*_(25)_ = −2.45; *p* < 0.05	16.6 ± 2.1	29.6 ± 2.8 #	*t*_(17)_ = −4.14; *p* < 0.05

*#p < 0.05 vs. respective control group. §p = 0.052 vs. respective control group.*

### Chronic Stress Promotes Anxiogenic-Like Behavior in Cagemate and Stress Groups, but Provoked Lower Weight Gain Only in Restrained Mice

Table 3 shows the weight gain measurements in the control (*n* = 44), cagemate (*n* = 42), and stress (*n* = 42) groups during 14 days of restraint stress. Statistical analysis indicated diminished body weight gain in the stress group compared to the control and cagemate groups [*F*_(2_,_127)_ = 80.91; *p* < 0.05].

**TABLE 3:** Body weight gain during 14 of restraint stress.

Group	Day		Weight gain (g)
	15th	28th	
Control	38.35 ± 0.53	47.28 ± 0.67	8.93 ± 0.42
Stress	36.86 ± 0.62	39.86 ± 0.56	3.00 ± 0.31*
Cagemate	36.79 ± 0.68	45.44 ± 0.66	8.65 ± 0.37

Data are presented as mean ± SEM. Control (n = 44), stress (n = 42), and cagemate (n = 42). One-way ANOVA.*p < 0.05 vs. control and cagemate groups.

**FIGURE 2:**
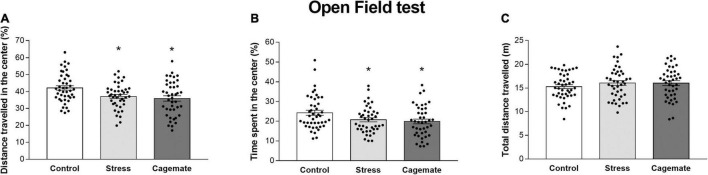
All data are presented as mean ± SEM. **(A)** Percentage of distance traveled, **(B)** percentage of time spent in the center of the open field, and **(C)** the total distance traveled (*n* = 42–44 per group) during the 5-min test. *p < 0.05 vs. control group. One-way ANOVA was followed by Newman-Keuls *post hoc* test.

Figure 2 and Table 4 present the anxiety-like behavior of control (*n* = 44), cagemate (*n* = 42), and stress (*n* = 42) groups tested in an open field 7 days after the last restraint stress session. One-way ANOVA followed by Newman–Keuls *post hoc* test revealed that cagemate and stress groups demonstrated a lower percentage of distance traveled (%DC) [*F*_(2_,_127)_ = 6.06; *p* < 0.05] and percentage of time spent (%TC) [*F*_(2_,_127)_ = 3.89; *p* < 0.05] in the center of the open field arena, but not the total distance traveled [*F*_(2_,_127)_ = 0.85; *p* = 0.43] during the test compared to the control. There is a difference in the absolute time spent (TC) [*F*_(2_,_127)_ = 3.97; *p* < 0.05], but not in absolute distance traveled (DC) [*F*_(2_,_127)_ = 1.54; *p* = 0.22] and number of entries (EC) [*F*_(2_,_127)_ = 1.91; *p* = 0.15] in the center of the apparatus (Table 4).

**TABLE 4:** Behaviors evaluated in the open field test.

Group	Behavior		
	EC	DC (m)	TC (s)
Control	47.55 ± 1.77	6.40 ± 0.24	72.82 ± 3.91
Stress	44.48 ± 2.03	5.96 ± 0.26	61.93 ± 3.08*
Cagemate	42.26 ± 1.99	5.76 ± 0.30	59.60 ± 3.63*

Data are presented as mean ± SEM. Control (n = 44), stress (n = 42), and cagemate (n = 42). *p < 0.05 vs. control group. One-way ANOVA. EC, number of entries in the center; DC, distance traveled in the center; TC, time spent in the center.

### Repeated Restraint Induces Locomotor Cross-Sensitization After Systemic Methamphetamine Administration in Cagemate and Stress Groups

**FIGURE 3:**
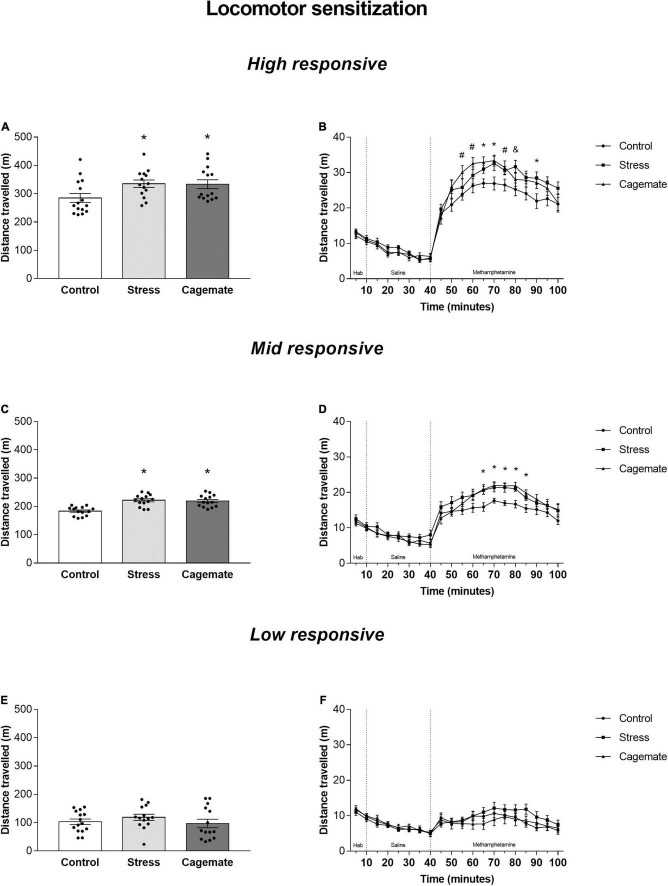
All data are presented as mean ± SEM. **(A)** Total distance traveled during 60 min test by high responsive mice after methamphetamine challenge (1.5 mg/Kg) (*n* = 14–15 per group); **(B)** distance traveled by high responsive mice during each 5-min block during habituation, saline challenge (1 mL/Kg), and methamphetamine challenge (1.5 mg/Kg) (*n* = 14–15 per group); **(C)** Total distance traveled during 60 min test by mid responsive mice after methamphetamine challenge (1.5 mg/Kg) (*n* = 14 per group); **(D)** distance traveled by mid responsive mice during each 5 min block during habituation, saline challenge (1 mL/Kg), and methamphetamine challenge (1.5 mg/Kg) (*n* = 14 per group); **(E)** total distance traveled during 60 min test by low responsive mice after methamphetamine challenge (1.5 mg/Kg) (*n* = 14–15 per group); **(F)** distance traveled by low responsive mice during each 5-min block during habituation, saline challenge (1 mL/Kg), and methamphetamine challenge (1.5 mg/Kg) (*n* = 14–15 per group); *p < 0.05 stress and cagemate vs. control group. #*p* < 0.05 cagemate vs. control group. &*p* < 0.05 stress vs. control group. One-way ANOVA followed by Newman–Keuls *post hoc* test. Differences between the groups were evaluated by planned comparisons in each 5 min block.

Figure 3 summarizes the locomotor activity of control, cagemate, and stress groups of high-, mid-, and low-responsive mice after administration of methamphetamine. One-way ANOVA followed by Newman–Keuls *post hoc* test showed that the cagemate and stress groups of high- [*F*_(2_,_40)_ = 3.78; *p* < 0.05, Figure 3A] and mid- [*F*_(2_,_39)_ = 17.54; *p* < 0.05, Figure 3C], but not low-responsive [*F*_(2_,_40)_ = 0.85; *p* = 0.43, Figure 3E] mice exhibited higher distance traveled during 60 min of locomotor cross-sensitization test compared to the respective control groups.

Figures 3B,D,F depict locomotor activity of all mice in 5-min blocks during habituation and after systemic administration of saline and methamphetamine. Planned comparisons indicated differences in some 5-min blocks for high responsive mice [55: cagemate vs. control (*p* < 0.05); 60: cagemate vs. control (*p* < 0.05); 65: stress and cagemate vs. control (*p* < 0.05); 70: stress and cagemate vs. control (*p* < 0.05); 75: cagemate vs. control (*p* < 0.05) and stress vs. control (*p* = 0.081); 80: stress vs. control (*p* < 0.05); 90: stress vs. control (*p* < 0.05) and cagemate vs. control (*p* = 0.054)], for mid-responsive mice [65: stress and cagemate vs. control (*p* < 0.05); 70: stress and cagemate vs. control (*p* < 0.05); 75: stress and cagemate vs. control (*p* < 0.05); 80: stress and cagemate vs. control (*p* < 0.05); 85: stress and cagemate vs. control (*p* < 0.05)], but not for low-responsive mice. One-way ANOVA for all 5-min blocks including habituation, saline, and methamphetamine periods may be seen in Table 5.

**TABLE 5:** One-way ANOVA for locomotor cross-sensitization behavior.

		Meth response		
Locomotor activity period	Blocks (minutes)	High responsive	Mid responsive	Low responsive
Habituation	5	*F*_(2_,_40)_ = 0.66; *p* = 0.53	*F*_(2_,_39)_ = 0.54; *p* = 0.59	*F*_(2_,_40)_ = 0.46; *p* = 0.64
	10	*F*_(2_,_40)_ = 0.31; *p* = 0.74	*F*_(2_,_39)_ = 0.10; *p* = 0.91	*F*_(2_,_40)_ = 0.35; *p* = 0.71
Saline	15	*F*_(2_,_40)_ = 0.22; *p* = 0.80	*F*_(2_,_39)_ = 0.99; *p* = 0.40	*F*_(2_,_40)_ = 0.42; *p* = 0.66
	20	*F*_(2_,_40)_ = 1.91; *p* = 0.16	*F*_(2_,_39)_ = 0.06; *p* = 0.94	*F*_(2_,_40)_ = 0.06; *p* = 0.94
	25	*F*_(2_,_40)_ = 1.23; *p* = 0.30	*F*_(2_,_39)_ = 0.29; *p* = 0.75	*F*_(2_,_40)_ = 0.19; *p* = 0.83
	30	*F*_(2_,_40)_ = 0.54; *p* = 0.59	*F*_(2_,_39)_ = 1.92; *p* = 0.16	*F*_(2_,_40)_ = 0.99; *p* = 0.38
	35	*F*_(2_,_40)_ = 1.01; *p* = 0.37	*F*_(2_,_39)_ = 1.32; *p* = 0.28	*F*_(2_,_40)_ = 0.09; *p* = 0.91
	40	*F*_(2_,_40)_ = 0.14; *p* = 0.87	*F*_(2_,_39)_ = 1.79; *p* = 0.18	*F*_(2_,_40)_ = 0.10; *p* = 0.91
Methamphetamine	45	*F*_(2_,_40)_ = 0.51; *p* = 0.61	*F*_(2_,_39)_ = 1.17; *p* = 0.32	*F*_(2_,_40)_ = 0.36; *p* = 0.70
	50	*F*_(2_,_40)_ = 1.63; *p* = 0.21	*F*_(2_,_39)_ = 1.13; *p* = 0.33	*F*_(2_,_40)_ = 0.05; *p* = 0.95
	55	*F*_(2_,_40)_ = 2.42; *p* = 0.10	*F*_(2_,_39)_ = 1.97; *p* = 0.15	*F*_(2_,_40)_ = 0.16; *p* = 0.85
	60	*F*_(2_,_40)_ = 2.39; *p* = 0.11	*F*_(2_,_39)_ = 2.27; *p* = 0.12	*F*_(2_,_40)_ = 0.94; *p* = 0.40
	65	*F*_(2_,_40)_ = 5.35; *p* < 0.05	*F*_(2_,_39)_ = 5.05; *p* < 0.05	*F*_(2_,_40)_ = 1.39; *p* = 0.26
	70	*F*_(2_,_40)_ = 3.87; *p* < 0.05	*F*_(2_,_39)_ = 5.36; *p* < 0.05	*F*_(2_,_40)_ = 1.14; *p* = 0.33
	75	*F*_(2_,_40)_ = 2.73; *p* = 0.077	*F*_(2_,_39)_ = 8.49; *p* < 0.05	*F*_(2_,_40)_ = 0.50; *p* = 0.61
	80	*F*_(2_,_40)_ = 2.98; *p* = 0.062	*F*_(2_,_39)_ = 8.87; *p* < 0.05	*F*_(2_,_40)_ = 0.86; *p* = 0.43
	85	*F*_(2_,_40)_ = 1.77; *p* = 0.18	*F*_(2_,_39)_ = 5.98; *p* < 0.05	*F*_(2_,_40)_ = 2.75; *p* = 0.076
	90	*F*_(2_,_40)_ = 3.48; *p* < 0.05	*F*_(2_,_39)_ = 1.22; *p* = 0.31	*F*_(2_,_40)_ = 1.34; *p* = 0.27
	95	*F*_(2_,_40)_ = 1.40; *p* = 0.26	*F*_(2_,_39)_ = 0.65; *p* = 0.53	*F*_(2_,_40)_ = 0.85; *p* = 0.44
	100	*F*_(2_,_40)_ = 1.41; *p* = 0.26	*F*_(2_,_39)_ = 1.41; *p* = 0.26	*F*_(2_,_40)_ = 0.40; *p* = 0.67

*The difference was significant for p-value less than 0.05 (p < 0.05).*

### Augmented Self-Grooming and Consolation-Like Behavior From Cagemate Toward Stressed Mice

**FIGURE 4:**
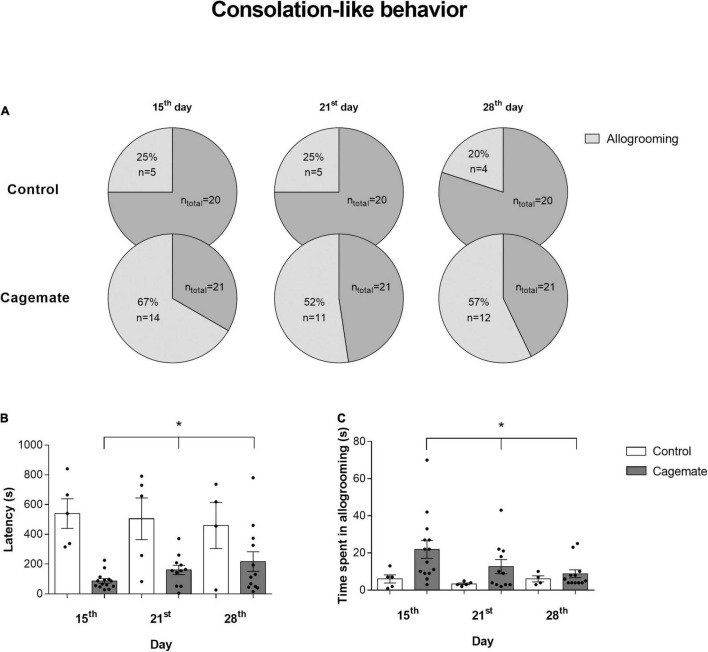
The data in the panels **(B,C)** are presented as mean ± SEM. **(A)** Percentage of subjects that exhibited consolation-like behavior in the 15th, 21st, and 28th experimental days in control and cagemate groups; **(B)** latency to start and **(C)** time spent in allogrooming behavior during 15 min. *p < 0.05 vs. control group. Two-way ANOVA followed by Newman-Keuls *post hoc* test.

Figure 4 depicts the consolation-like behavior evaluated through the percentage of subjects who exhibited allogrooming behavior (Figure 4A), latency of first allogrooming episode (Figure 4B), and time spent doing allogrooming (Figure 4C) from control and cagemate groups toward their conspecifics in each evaluated day. Statistical analysis of the sample proportion revealed differences in the percentage of subjects displaying allogrooming between control and cagemate groups on the 15th and 28th day. In the first and last stress days (15th and 28th days) the prevalence of animals showing allogrooming is greater in the cagemate group (*p* < 0.05). In the control group, the prevalence of allogrooming was 25% (*n* = 5) on the 15th and 20% (*n* = 4) on the 28th day, while this prevalence among stressed animals was 66.7% (*n* = 14) on the 15th and 57.1% (*n* = 12) on the 28th day. On the 21st day, the proportion of mice exhibiting allogrooming behavior was 25% (*n* = 5) in the control group vs. 52.4% (*n* = 11) in the cagemate group (*p* = 0.11). Two-way ANOVA indicated a significant effect of stress [*F*_(1_,_45)_ = 5.98; *p* < 0.05], but not the day factor [*F*_(2_,_45)_ = 1.28; *p* = 0.29]. There was no influence of interaction between the factors [*F*_(2_,_45)_ = 0.99; *p* = 0.38] at the time of allogrooming. For the latency to start allogrooming, statistical analysis showed the influence of stress [*F*_(1_,_45)_ = 33.76; *p* < 0.05], but not the day factor [*F*_(2_,_45)_ = 0.06; *p* = 0.94]. There was no effect of interaction between the factors [*F*_(2_,_45)_ = 1.02; *p* = 0.37] in the latency of the beginning of the consolation-like behavior. Newman–Keuls *post hoc* test revealed that the cagemate group started allogrooming toward stressed conspecifics in less time than the control in all days assessed (*p* < 0.05). Together, these data suggest that cohabitation with a partner subjected to chronic stress induces increased consolation-like behavior, which is seen as augmented time spent in allogrooming and diminished latency to begin this behavior.

Table 6 shows time spent in self-grooming of control (*n* = 20) and cagemate (*n* = 21) groups measured on the 15th, 21st, and 28th days at the end of restraint stress sessions. Two-way ANOVA indicated influence of stress [*F*_(1_,_117)_ = 23.63; *p* < 0.05] and day factors [*F*_(2_,_117)_ = 4.41; *p* < 0.05]. Statistical analysis also revealed a strong trend in the interaction between stress and day factors [*F*_(2_,_117)_ = 2.94; *p* = 0.057] on self-grooming behavior. Newman–Keuls *post hoc* test demonstrated that cagemate spent more time doing self-grooming behavior than the control group on the 15th day (*p* < 0.05), almost on the 21st day (*p* = 0.058), but not on the 28th day (*p* = 0.49). Inside the cagemate group, self-grooming behavior on the 15th day is higher than the 21st and 28th days evaluated (*p* < 0.05). Cagemate group spent more in self-grooming behavior on the 15th day than any day from the control group (*p* < 0.05), on the 21st day compared to the 28th day (*p* < 0.05), and almost to 15th day from the control group (*p* = 0.064).

**TABLE 6:** Self-grooming measured in three days during post-stress period.

	Day		
Self-grooming (s)	15th	21st	28th
Control	46.80 ± 13.48	35.70 ± 7.16	38.00 ± 4.53
Cagemate	161.38 ± 28.25^#^*	103.86 ± 24.08^a^^b^^c^	66.57 ± 13.80

Data are presented as mean ± SEM. Control (n = 20) and cagemate (n = 21). One-way ANOVA. *p < 0.05 vs. respective control group. ^#^p < 0.05 vs. control in any day.

*^a^p = 0.064 vs. control group on the 15th day.*

*^b^p = 0.057 vs. control group on the 21st day.*

*^c^p < 0.05 vs. control group on the 28th day.*

## Discussion

In the present study, we observed that restraint stress for 14 days added to 14 days to establish familiarity is necessary for the development of anxiogenic-like behaviors displayed by cagemates. Furthermore, we found that witnessing a conspecific subjected to chronic restraint stress for 14 days induced anxiety-like behavior in the open-field test and promoted locomotor cross-sensitization to methamphetamine in high- and mid-responsive mice. Lastly, we demonstrated that cagemates exhibited higher consolation behavior after stress sessions than the control group.

In the current study, we found that the anxiogenic effects exhibited by cagemates depend on the degree of familiarity since emotional contagion was observed after cohabitation for 14 days. Langford et al. (2006) have already shown the importance of familiar bounds in the sensitivity of visceral pain. Specifically, they demonstrated that siblings displayed enhanced abdominal writhes compared to strangers when subjected to intraperitoneal acetic acid administration. This empathy-related behavior was observed only after at least 14 days of living together (Langford et al., 2006). Following the findings of the Langford group, several studies replicated the influence of familiarity on the social modulation of abdominal pain (Martin et al., 2015; Lu et al., 2018). In another context, subjects observing siblings, sexual mates, or same-sex cagemates receiving footshocks froze more than the group witnessing strangers in suffering (Jeon et al., 2010; Gonzalez-Liencres et al., 2014). Moreover, rodents may develop fear conditioning through the observation of conspecifics that undergo tone-paired footshock, an approach known as observational fear learning. In this case, siblings displayed more freezing than strangers after tone presentation (Lidhar et al., 2017; Pisansky et al., 2017). Interestingly, empathy-related behaviors are not exclusively for familiar individuals since empathy may be increased by the previous distress experience of the observer (Luo et al., 2020). As proposed by Preston and de Waal (2002), the behaviors of the subject (observer) are automatically and unconsciously driven by the same neural substrates activated in the object (demonstrator), inducing the representation of the resembling emotional states. The more similar and socially bounded, the greater is the identification of the subject with the object, which augments the matching of the behavioral and autonomic responses (de Waal, 2008).

Confirming previous results from our group, we found that living with conspecifics subjected to repeated restraint stress increases anxiety-like behavior in cagemates, as well as directly stressed mice tested in the open field (Carneiro de Oliveira et al., 2017). Moreover, our previous findings also reported emotional contagion in mice provoked by neuropathic pain. In these studies, cohabitation with a mouse subjected to chronic pain diminished the exploration of open arms in the EPM (Baptista-de-Souza et al., 2015; Carmona et al., 2016; Benassi-Cezar et al., 2020) and caused hypersensitivity to visceral pain (Baptista-de-Souza et al., 2015, 2021; Zaniboni et al., 2018; Tavares et al., 2021). Taken together, our data corroborate studies from literature showing emotional contagion through approaches that the cagemate witnesses or shares aversive stimuli.

For instance, studies have reported that mice or rats observing traumatic events, in this case conspecifics subjected to repeated social defeat, elicited enhanced avoidance of open arms (Sial et al., 2016; Kochi et al., 2017), affecting (Patki et al., 2014, 2015), or not (Warren et al., 2013), the general locomotor activity in the open field. Additionally, Miao et al. (2018) found decreased exploratory activity of spectator pregnant mice in the open arms after successive exposure to mate social defeat. Conversely, Li et al. (2020) showed that mandarin voles witnessing partners subjected to chronic social defeat did not display changes in the time spent in the central area and the total distance traveled by the open field.

Regarding empathy for pain, a body of evidence, including data from our group, has shown the effects of emotional contagion of pain-promoting alterations in anxiety-like behaviors of cagemates. Using a model of chronic neuropathic pain, researchers from our laboratory demonstrated anxiogenic-related behaviors in mouse cages tested in EPM and open fields (Baptista-de-Souza et al., 2015; Carmona et al., 2016; Benassi-Cezar et al., 2020). In approaches where cagemates live with conspecifics in other kinds of pain conditions, such as melanoma-bearer mice (Tomiyoshi et al., 2009), formalin-induced paw inflammation (Parent et al., 2012; Mohammadi et al., 2019; Nazeri et al., 2019), and neuropathy (Wallace et al., 2008) augmented anxiety through tests conducted in EPM and open field models was also observed. These results reinforce the idea of observational contagion in rodents through the ability to recognize the negative emotional state of a conspecific.

Chronic restraint stress increased the acute psychomotor effects of methamphetamine in both stress and control groups. Several studies have shown cross-sensitization between repeated restraint stress and psychostimulants, such as cocaine (Lepsch et al., 2005) and amphetamine (Deroche et al., 1992; Kabbaj et al., 2002; Doremus-Fitzwater and Spear, 2010; Cruz et al., 2012; Carneiro de Oliveira et al., 2016). However, no previous studies have investigated the vicarious consequences of restraint in psychostimulant-induced locomotor behavior. Interestingly, Garcia-Carachure et al. (2020) assessed the influence of witnessing chronic social defeat stress in cocaine-conditioned place preference and observed a higher preference for cocaine-paired place in vicarious-stressed group when compared to non-stressed controls. Thus, these findings indicate that vicarious stress as direct exposure to harmful situations may modify drug effects and induce seeking behaviors.

We also found a higher consolation behavior from cagemates toward their stressed partners than the control group. We evaluated the latency for the beginning of allogrooming and the time spent in allogrooming on the 1st, 7th, and 14th days of stress sessions. Our findings demonstrated that the cagemate group reduced the latency to start allogrooming and enhanced the duration of this consolation-like behavior. Previous studies have reported that the interaction with a cagemate in a distress situation enhances the consolation behavior (Knapska et al., 2010; Burkett et al., 2016; Li et al., 2018, 2019; Lu et al., 2018; Kiyokawa et al., 2019; Du et al., 2020). In these studies, the results demonstrated enhanced allogrooming from observers toward their mate demonstrators subjected to social defeat stress procedure (Li et al., 2019) or footshock (Knapska et al., 2010; Burkett et al., 2016; Kiyokawa et al., 2019). Furthermore, studies have shown that observers diminish the latency to start and enhance allogrooming toward demonstrators in pain situations (Li et al., 2018; Lu et al., 2018; Du et al., 2020). Taken together, these findings highlight the concern of observers with conspecific distress conditions.

Interestingly, mice from the control group, even being lower than the cagemate group, exhibited allogrooming toward their partners in the absence of stress or separation. This behavior was unexpected since the mice from the control group had no stimulus to trigger allogrooming. In contrast, some studies showed engagement in consolation-like behavior in control groups after a brief separation of their partners (Knapska et al., 2010; Burkett et al., 2016; Li et al., 2019). Moreover, although the control group has not been submitted to any procedure, the mice were moved to the test chamber probably inducing an emotional arousal by novel environment (Li et al., 2018; Lu et al., 2018; Du et al., 2020). In our case, although it is merely speculative, we believe that the allogrooming exhibited by the control group was provoked by the transference of the mice from the animal facility to the test room. Thus, more studies should be conducted to answer this important question.

Previous studies assessing the behavioral mechanisms of consolation usually evaluated the influence of acute aversive stimuli on allogrooming. Thus, our data investigating the consolation-like behavior over the stress period has no precedent in the literature. Note that the consolation was inefficient in preventing the development of anxiety-like behavior and locomotor sensitization, corroborating the findings of Li et al. (2019). This process, also known as social buffering, in some cases can prevent or reverse the expression of anxiety-like behavior induced by aversive stimuli (Burkett et al., 2016; Kiyokawa et al., 2019). Therefore, due to its relevance, continued research focusing on the consequences and motivations of consolation behavior is needed.

Surprisingly, although statistical analysis did not indicate differences among the days of measurements, visually we may see a tendency to decrease the time in consolation as well as increase the latency through the subsequent days. The data obtained from self-grooming show that the time engaged in this behavior declines over time. It is well-established that self-grooming, in some situations, may denote augmented stress conditions (Fernández-Teruel and Estanislau, 2016; Kalueff et al., 2016; Song et al., 2016) inciting us to suggest a habituation to repeated stress sessions by cagemates. Thus, based on self-grooming results, we could extrapolate this behavior adaptation to allogrooming, indicating a coping process exhibited by cagemates without affecting anxiogenesis. Although plausible, this hypothesis needs to be confirmed through further investigations.

In conclusion, we observed the presence of emotional contagion in familiar mice through increased anxiety behavior in both stress and cagemate groups. It is important to highlight that cohabitation with a partner in a harmful situation should not be analyzed as a simple psychological stress, but needs to be viewed as a complex process that demands perception of the aversive condition, identification of negative emotional state from another, and engagement in relieving conspecific distress. The consolation behavior reveals an emotional arousal that motivates the cagemate to display prosocial behavior. Therefore, our findings demonstrate that empathy-based concernments directed to a familiar conspecific in distress conditions may provoke psychological disturbances and augmented drug seeking.

## Data Availability Statement

The original contributions presented in the study are included in the article/supplementary material, further inquiries can be directed to the corresponding author.

## Ethics Statement

All procedures were performed in accordance with the recommended protocol approved by the Brazilian Guidelines for Care and Use of Animals for Scientific and Educational Purposes, elaborated by the National Council of Control of Animal Testing (CONCEA). The animal study was reviewed and approved by Comissão de Ética no Uso de Animais Universidade Federal de São Carlos (CEUA 4996150816).

## Author Contributions

PCdO and IC: conceptualization, methodology, data curation, formal analysis, investigation, project administration, writing—original draft preparation, and writing—review and editing. MC: data curation, formal analysis, investigation, project administration, and writing—review and editing. LS and AO: data curation, formal investigation, and writing—review and editing. AC-d-S: conceptualization, funding acquisition, project administration, writing—review and editing, and supervision. All authors contributed to the article and approved the submitted version.

## Conflict of Interest

The authors declare that the research was conducted in the absence of any commercial or financial relationships that could be construed as a potential conflict of interest.

## Publisher’s Note

All claims expressed in this article are solely those of the authors and do not necessarily represent those of their affiliated organizations, or those of the publisher, the editors and the reviewers. Any product that may be evaluated in this article, or claim that may be made by its manufacturer, is not guaranteed or endorsed by the publisher.
